# The influence of personality traits and facets on visuo-spatial task performance and self-assessed visuo-spatial inclinations in young and older adults

**DOI:** 10.1371/journal.pone.0220525

**Published:** 2019-08-05

**Authors:** Elena Carbone, Chiara Meneghetti, Erika Borella

**Affiliations:** Department of General Psychology, University of Padova, Padova, Italy; University of L'Aquila, ITALY

## Abstract

**Aims:**

Personality traits are suggested to influence adults’ cognitive performance, but little is known about their association with visuo-spatial competence, in terms of those visuo-spatial abilities and inclinations crucial to remaining autonomous, especially with aging. This study newly investigated whether, and to what extent, major traits and narrower facets of personality influence young and older adults’ performance in the so-called objective visuo-spatial abilities (mental rotation and visuo-spatial working memory [VSWM]), and self-assessed visuo-spatial inclinations (pleasure and anxiety in exploring places).

**Method:**

Seventy young adults (18–35 years old) and 70 older adults (65–75 years old) completed the Big-Five questionnaire, objective rotation and VSWM tasks, and spatial self-assessments on pleasure and anxiety in exploring places.

**Results:**

Hierarchical regression models confirmed that age negatively predicted the variance in objective visuo-spatial tasks, but not in self-assessed visuo-spatial inclinations, while only the latter were slightly influenced by gender (in favor of men). Further, both objective visuo-spatial abilities (albeit modestly) and self-assessed visuo-spatial inclinations were predicted by higher Conscientiousness. The latter were also predicted by higher Emotional Stability. Finally, a better objective visuo-spatial performance was explained (again modestly) by lower Dynamism and Politeness, and higher Emotion Control, while higher Perseverance, Emotion Control and Cooperativeness explained a moderate part of the variance in the positive self-assessed visuo-spatial inclinations.

**Conclusions:**

Our findings indicate that, beyond age and gender, some personality traits and facets predict self-assessed visuo-spatial inclinations to a larger extent than objective visuo-spatial performance. These results are discussed within the spatial cognition and aging framework.

## Introduction

Personality traits refer to an individual’s consistent pattern of thoughts, feeling and actions [1]. According to the dominant theoretical framework of the Big Five model [2], personality can be divided into five major traits–Energy (or Extraversion), Conscientiousness, Emotional Stability, Agreeableness, and Openness. Each of these traits is also defined by narrower characteristics, namely facets. A significant body of evidence shows that personality is related to relevant life outcomes (e.g., academic and occupational achievement, quality of interpersonal relationships, physical and psychological health, and longevity) [3,4], as well as to general intellectual abilities (i.e., crystallized intelligence, fluency and knowledge) [5,6]. Given the involvement of personality in cognitive functioning, there is growing interest in understanding to what extent it can influence cognitive performance with aging [7,8]. Although personality traits are generally conceived as fairly stable individual characteristics [9], there is evidence of them changing over the life span: Extraversion tends to decrease, Openness peaks in adolescence and only declines in later adulthood (in people over 75 years old, the so-called ‘fourth age’ [10,11]), while Conscientiousness, Emotional Stability, and Agreeableness increase over time.

Studies involving healthy older adults have also demonstrated an association between personality traits (measured using the Big Five model) and cognition, either in terms of both general intellectual abilities or global cognitive functioning, or in certain specific cognitive domains, such as verbal cognitive ability, memory functioning [7,8,12] and fluid intelligence -this latter assessed using reasoning and spatial visualization tasks [13–20]-. The associations between personality and cognition are generally modest in older adults, however [9,10], and the most consistent findings point to higher Openness and Conscientiousness, and lower Neuroticism correlating positively with a better cognitive functioning, as in adulthood, and with a slower age-related cognitive decline [7,8]. In fact, older adults scoring higher for Openness and Conscientiousness, for instance, seem more likely to engage in cognitively stimulating activities (e.g., reading newspapers, learning to use a computer) that have been found to positively affect cognitive functioning, and they are more likely to adopt a healthy lifestyle that protects against the effects of cognitive aging [8]. In contrast, Neuroticism (i.e., lower Emotional Stability) has been indicated as a risk factor for cognitive decline: individuals scoring higher for Neuroticism seem less able to control their own negative emotional reactions (such as anger or anxiety), and are therefore more exposed to the detrimental effects of anxiety and stress, which can negatively affect cognitive performance [7,8].

It is worth noting that we still know little about the relationship between personality traits and other cognitive abilities, such as those crucial to individuals’ environment learning accuracy, and those known to be important in enabling older adults to remain autonomous, like visuo-spatial competence [21–23]. According to the spatial cognition framework [24], visuo-spatial competence can be divided into the visuo-spatial abilities needed to generate, retain and process visuo-spatial information (measured with objective tasks), which can be called objective visuo-spatial abilities [25], and spatial self-reports (measured with questionnaires), which can be described as self-assessed visuo-spatial inclinations [26]; and the two are considered as distinct, though related factors [24]. The objective visuo-spatial abilities include both higher-order visuo-spatial skills, such as mental rotation (the ability to turn two- or three-dimensional objects in one’s mind, or to adopt different views [27]), and processing abilities like visuo-spatial working memory (VSWM; the ability to retain and process visuo-spatial information [28]), which can be grouped together and considered as a single visuo-spatial factor [24].

The few studies conducted on young adults indicated that personality traits are only weakly related to objective visuo-spatial abilities [12,29,30], as measured with paper-and-pencil tasks (like the Mental Rotations Test [31]) [29,30], or using classical VSWM laboratory-based measures (like the Corsi Block-tapping Test [32], or the Pathways Span task [33]) [30], or assessed using verbal, visuo-spatial and updating tasks [12]. As for aging studies, to our knowledge, the association between personality traits and visuo-spatial abilities has only been investigated by Schaie, Willis and Caskie [17]. They found that higher Openness and lower Agreeableness predicted a better spatial rotation performance in individuals from 22 to 84 years old, though accounting for a very small part of the variance (6%).

As for self-assessed visuo-spatial inclinations, these encompass pleasure in exploring, for example (i.e. attitudes to, and feelings about visiting places), and spatial anxiety (i.e. worrying about getting lost), which respectively reflect positive and negative inclinations to approaching the environment [26]. Previous studies on young adults showed that such self-assessed visuo-spatial inclinations are related to personality traits and facets, but slightly differently: pleasure in exploring correlated positively with Extraversion (the Dynamism and Dominance facets), Openness (the Openness to Experience facet), and Conscientiousness (the Perseverance facet), while spatial anxiety correlated negatively with Extraversion (Dynamism), Openness (Openness to Experience), Emotional stability (Emotion Control), and Conscientiousness (Scrupulousness) [30]. No studies have yet explored the association between personality and self-assessed visuo-spatial inclinations in older adults, however.

In short, while the association found between personality and objective visuo-spatial abilities—addressed in a few studies (in young adults [12,29,30]; in older adults [17])—has proved weak, a moderate association between personality and self-assessed visuo-spatial inclinations has emerged, though only in young adults [29,30]. Such a relationship between personality and visuo-spatial competence has yet to be clarified, and thus merits further investigation.

The present study thus aimed to newly investigate whether, and to what extent, young and older adults’ personality dispositions are associated with their visuo-spatial competence, in terms of their objectively-measurable visuo-spatial abilities and their subjectively-assessed visuo-spatial inclinations. Using the Big Five model [2], we considered the five major personality traits and their narrower facets, in an effort to better conceptualize the association between personality and visuo-spatial competence [7].

Since objective visuo-spatial abilities are known to be sensitive to the effects of aging [34,35], whereas self-assessed visuo-spatial inclinations have been found fairly stable over time [26,34], we expected to confirm the impairments in older adults in objective visuo-spatial tasks performance, but not in self-assessed visuo-spatial inclinations [26,34,35]. Different patterns of associations might then be expected between personality traits (and facets) and objective visuo-spatial abilities on one hand, and self-assessed visuo-spatial inclinations on the other hand. In particular, for objective visuo-spatial abilities we would expect to find, in line with previous studies in young [29,30] and older adults [7,13–20], some (albeit modest) associations between most major personality traits and performance on visuo-spatial tasks. For the self-assessed visuo-spatial inclinations, not impaired by increasing age [26,34], on the other hand, we would expect to find stronger associations with most personality traits, as previously suggested, in young adults at least [29,30].

As for personality facets, we might expect them to mirror the pattern of associations found between major personality traits and visuo-spatial competence. However, since it has been suggested that these narrower dimensions of personality are not simply interchangeable with the broad traits they are designed to reflect [10,36–38], we might also expect some different patterns of associations with objective visuo-spatial abilities and self-assessed visuo-spatial inclinations to emerge (compared with those seen for major personality traits).

Finally, gender was considered in the analyses too, given the well-known role of gender in influencing visuo-spatial abilities and inclinations [25,39,40].

## Materials and methods

The study was approved by the local Ethics Committee (protocol number 2370). All participants were given information about the study and gave their written informed consent in accordance with the Declaration of Helsinki (World Medical Association, 2013).

### Participants

Seventy young adults (age range: 18–34 years; *M* = 23.51, *SD* = 2.95; 36 females) and 70 older adults (age range: 65–75 years; *M* = 68.63, *SD* = 3.29; 41 females) volunteered for the study. All participants were Italian mother-tongue community dwellers recruited through associations in north-eastern Italy, or by word of mouth. The inclusion criteria were: (i) a good state of physical and mental health, and no history of psychiatric or neurological disorders, ascertained by means of a semi-structured interview [41]; and, for older adults, (ii) a score in the Mini-Mental State Examination [42] above the cut-off of 27. The two age groups did not differ in terms of educational level, *F*_(1,138)_ = 1.94; *p* = .16, *d* = .23, (young adults: *M* = 12.74, *SD* = 0.65; older adults: *M* = 12.50, *SD* = 1.31), or gender distribution (χ^*2*^ = .72, *p* = .39).

### Materials

#### Personality

Big Five Questionnaire-60 (BFQ); [43]. This is a short version containing the 60 items with the best psychometric properties from the BFQ [44], with good reliability (Cronbach’s alpha from .70 for Energy to .85 for Emotional Stability, and from .53 for Cooperativeness to .81 for Emotion Control). Participants had to indicate the extent to which each of the 60 statements described “how they are” as a person on a 5-point scale from 1 (very false for me) to 5 (very true for me). The answers give rise to five domain scales, i.e., Energy (or Extraversion), Agreeableness, Conscientiousness, Emotional Stability, and Openness, and their related facets, which are respectively Dynamism and Dominance, Cooperativeness and Politeness, Scrupulousness and Perseverance, Emotion Control and Impulse Control, Openness to Culture and Openness to Experience.

#### Rotation ability tasks

Short Mental Rotations Test [sMRT; 26]. This task involves identifying two of four abstract 3D objects that match a target object in a rotated position, and has shown good reliability (Cronbach’s alpha = .81). Participants are shown 10 items to complete in 5 min. The final score–the dependent variable–is the number of correct answers (Score: minimum 0—maximum 10).

Short Object Perspective Test [sOPT; 26]. This task involves looking at a layout of seven objects and imagining standing alongside one object, facing another, and pointing to a third. The layout remains visible to participants and a circle is used to provide the answer. Participants have to complete 6 items with a time constraint of 5 min. The absolute angle of error is calculated for each item in degrees of difference between the correct angle and the one indicated by participants. This task has shown good reliability (Cronbach’s alpha = .80). The final score–the dependent variable–is the mean error (in degrees; range 0°-180°), so higher scores correspond to a worse performance.

#### Visuo-spatial working memory tasks

Backward Corsi Blocks Test [CBT; 32]. In this task the experimenter taps sequences of blocks (3 x 3 x 3 cm) placed at random on a board (30 x 25 cm), at a rate of one block every 2 sec. Then participants are asked to tap the same sequences of blocks, but in reverse order. The number of blocks in a sequence gradually increases in length (from 2 to 9 blocks). This measure has shown good reliability (Cronbach’s alpha = .94). The final score–the dependent variable–is the number of blocks in the longest sequence correctly recalled (minimum 2—maximum 9).

Jigsaw Puzzle test [Puzzle; 41]. This task consists of 27 drawings of common objects, each broken down into 2 to 10 numbered pieces forming a puzzle. Each whole drawing is presented together with a corresponding verbal label for 2 sec, then it is hidden. Participants are shown the numbered pieces and asked to complete the puzzle mentally by indicating where the pieces should go on a blank matrix, without actually moving them (time constraint for each figure: 90 sec). The complexity of the task varies, depending on the number of pieces forming a given figure (from 2 to 10). This task has shown good reliability (Cronbach’s alpha = .83). The final score–the dependent variable–is the sum of the levels corresponding to the three most difficult puzzles correctly completed.

Pathways Span Task [PST; 33]. This is a computerized version of the original task, in which a matrix with a colored cell in the bottom left corner is presented on the screen for 4 sec. The initial stimulus is then removed, and participants are asked to mentally imagine moving along a path on the matrix (starting from the colored cell) by following directions (move forwards, backwards, left, or right) given by means of arrows (up, down, left, right) that appear on the screen one at a time for 2 sec each. At the end, a blank matrix appears and participants are asked to indicate the final position they reached. The difficulty of the task varies depending on the size of the matrix (from 2 x 2 to 6 x 6 cells) and the length of the path (the number of displacements indicated by the arrows). This task has shown good reliability (Cronbach’s alpha = .85). The final score–the dependent variable–is the sum of the levels corresponding to the three most complex trials correctly completed.

#### Self-assessed visuo-spatial inclinations scales

Attitudes to Orientation Tasks scale [AtOT; 26]. This scale comprises 10 items assessing two factors: (i) pleasure in exploring places (e.g., “I like to find new ways to reach familiar places”; 5 items); and (ii) no pleasure in exploring places (e.g., “When I see a new road, I avoid taking it because I don’t know where it ends”; 5 items), with good reliability (Cronbach’s alpha from .78 to .83). Participants rate each item on a Likert scale from 1 (not at all) to 6 (very much). The score is calculated as the reverse of the rating on the items indicating no pleasure in exploring, so the final score expresses the respondent’s pleasure in exploring (minimum 10—maximum 60), and higher scores indicate greater pleasure in exploring places.

Spatial Anxiety scale [SA; 26]. This scale comprises 8 items assessing the degree of space-related anxiety experienced in an environment (e.g., “Going to an appointment in an unfamiliar part of the city”), and has shown good reliability (Cronbach’s alpha = .87). Answers are given on a Likert scale from 1 (not at all) to 6 (very much). The final score is the sum of the ratings for each item (minimum 8—maximum 48), with higher scores corresponding to greater spatial anxiety.

### Procedure

Participants attended two individual sessions of approximately 60 min each, conducted by a trained examiner. At the start of the first session, after signing an informed consent form, all participants completed a general information questionnaire (and the older participants completed the MMSE). Then, the visuo-spatial tasks and the self-assessments were administered in two sets: one included the sMRT, backward CBT, PST and AtOT; the other the Puzzle, sOPT, and BFQ. The order of presentation was the same for all participants within each set, but the order of the sets was counterbalanced between participants and administered at either the first or the second individual session. The SA scale was always presented at the end of the second session to avoid any activation of negative mood.

### Statistical analyses

First, a factor analysis was run, including the two mental rotation tasks, the three VSWM tasks, and the two spatial self-assessments to establish whether the visuo-spatial measures considered represent two distinct aspects of visuo-spatial competence, i.e., objective visuo-spatial abilities (VSWM and rotation abilities) and self-assessed visuo-spatial inclinations. Principal components factor extraction with Promax rotation was used, applying Kaiser’s eigenvalue greater than one rule as well as Horn’s [45] parallel analysis method to derive common underlying factors.

Then Pearson’s correlations were run between all the measures of interest.

Finally, to elucidate the association between personality and visuo-spatial competence, hierarchical regression analyses were conducted with demographic characteristics, i.e. age and gender (the latter given its influence on visuo-spatial competence), added in Step 1, and the major personality traits, added in Step 2, as predictors of objective visuo-spatial abilities and self-assessed visuo-spatial inclinations (dependent variables), respectively. Then other regression analyses were run, but in this case the narrower facets of personality were added as predictors in Step 2. All the models were checked for outliers (Cook's distance <1).

## Results

The raw scores for personality traits and facets were considered in our analyses. Descriptive statistics for the measures of interest are presented in Table 1.

**Table 1:** Means (*M*) and standard deviations (*SD*) of objective visuo-spatial abilities and self-assessed visuo-spatial inclinations’ measures, and personality traits and facets, by age group.

	Young adults (*n* = 70; 36 females)		Older adults (*n* = 70; 41 females)	
	*M*	*SD*	*M*	*SD*
Objective visuo-spatial abilities				
Rotation abilities:				
short Mental Rotations Test	4.39	2.56	2.33	1.67
short Object Perspective Test	32.69	24.49	52.39	36.56
Visuo-spatial working memory:				
Backward Corsi Blocks Test	5.26	0.94	4.47	0.76
Jigsaw Puzzle test	22.00	4.39	17.91	4.19
Pathways Span Task	24.64	4.82	18.90	5.09
Self-assessed spatial inclinations:				
Attitude to Orientation Tasks scale	34.04	7.27	34.20	8.42
Spatial Anxiety scale	22.29	6.43	21.77	6.58
Personality:				
ENERGY	3.16	0.49	3.03	0.44
Dynamism	3.41	0.67	3.29	0.69
Dominance	2.92	0.62	2.78	0.56
CONSCIENTIOUSNESS	3.52	0.57	3.51	0.55
Scrupulousness	3.52	0.64	3.47	0.64
Perseverance	3.52	0.69	3.55	0.61
EMOTIONAL STABILITY	2.90	0.78	3.25	0.54
Emotion Control	2.91	0.90	3.26	0.61
Impulse Control	2.89	0.87	3.24	0.68
AGREEABLENESS	3.35	0.47	3.27	0.52
Cooperativeness	3.52	0.58	3.33	0.58
Politeness	3.19	0.57	3.21	0.57
OPENNESS	3.33	0.51	3.46	0.58
Openness to Culture	3.11	0.67	3.53	0.68
Openness to Experience	3.56	0.68	3.40	0.65

### Factor structure of visuo-spatial competence

The Kaiser-Meyer-Olkin measure of sampling adequacy was .73 (above the commonly recommended value of .60 [46]), and Bartlett’s test of sphericity was significant (χ^2^_(21)_ = 247.10, *p* < .001). Both Kaiser’s eigenvalue criterion and Horn’s parallel analysis confirmed that two factors, which explained 62.28% of the variance in the model (*r* = .14), should be retained. The two mental rotation and three VSWM measures loaded strongly on the first factor (see Table 2), which explained 39.46% of the variance (eigenvalue = 2.76), and could be interpreted as expressing objective visuo-spatial abilities. The second factor, which explained 22.80% of the variance (eigenvalue = 1.59), and the two spatial self-assessments loaded strongly on it (see Table 2), was interpreted as referring to self-assessed visuo-spatial inclinations.

**Table 2:** Results of factor analysis (Promax rotation method) for measures of objective visuo-spatial abilities and self-assessed visuo-spatial inclinations.

	Objective visuo-spatial abilities	Self-assessed visuo-spatial inclinations
short Mental Rotations Test	**.64**	.32
short Object Perspective Test	**-.75**	.06
Backward Corsi Blocks Test	**.75**	-.06
Jigsaw Puzzle test	**.73**	.11
Pathways Span Task	**.77**	-.20
Attitude to Orientation Task scale	-.10	**.88**
Spatial Anxiety scale	-.04	**-.84**

Note. *n* = 140

Significant values are shown in bold type.

Factor loadings higher than .50 (in bold) were used to interpret the factors.

In the light of the results of factor analysis, and considering the moderate-to-large correlations between the objective visuo-spatial abilities, and the large correlation between the two spatial self-assessments (see S1 Table), two composite scores were then calculated, one by averaging the *z*-scores from the two rotation ability measures and the three VSWM tasks, the other by averaging the *z*-scores from the two self-assessment scales of visuo-spatial inclinations. These composite scores were used in subsequent analyses as measures of objective visuo-spatial abilities and self-assessed visuo-spatial inclinations, respectively.

### Relation between personality and visuo-spatial competence

#### Correlations

Age showed a large negative correlation with objective visuo-spatial abilities, while no significant correlations emerged between age and self-assessed visuo-spatial inclinations (see Table 3). Small positive correlations were also found between age and: (i) the Emotional Stability trait (and both its facets, Emotion Control and Impulse Control); (ii) the Openness to Culture facet of the Openness trait.

**Table 3:** Correlation matrix for all measures of interest.

	1	2	3	4	5	6	7	8	9	10	11	12	13	14	15	16	17	18
1. Age	-																	
2. Gender^a^	-.07																	
3. Objective visuo-spatial abilities	-.59**	.15																
4. Self-assessed visuo-spatial inclinations	.04	.37**	.12															
5. ENERGY	-.11	-.11	-.08	.14														
6. Dynamism	-.07	-.30**	-.17*	.02	.77**													
7. Dominance	-.09	.15	.06	.19*	.68**	.07												
8. CONSCIENTIOUSNESS	-.01	-.01	.08	.40**	.23**	.23**	.10											
9. Scrupulousness	-.03	.04	.11	.31**	.12	.12	.05	.86**										
10. Perseverance	.02	-.05	.03	.38**	.27**	.26**	.12	.86**	.48**									
11. EMOTIONAL STABILITY	.26**	.16*	-.01	.34**	-.06	-.15	.07	.07	.06	.06								
12. Emotion control	.23**	.19*	.04	.41**	.01	-.15	.19*	.06	-.00	.12	.87**							
13. Impulse control	.22**	.10	-.06	.19*	-.12	-.11	-.05	.06	.12	.00	.87**	.53**						
14. AGREEABLENESS	-.08	-.19*	-.06	.19*	.35**	.51**	-.02	.28**	.19*	.29**	.01	-.02	.04					
15. Cooperativeness	-.15	-.17*	.05	.23**	.41**	.56**	.00	.37**	.27**	.37**	-.03	-.07	.01	.86**				
16. Politeness	.02	-.13	-.17*	.10	.19*	.31**	-.05	.10	.05	.13	.06	.04	.07	.85**	.48**			
17. OPENNESS	.11	-.23**	-.15	.14	.27**	.36**	.01	.31**	.23**	.30**	.15	.11	.15	.40**	.39**	.29**		
18. Openness to culture	.30**	-.10	-.19*	.17*	.13	.13	.06	.26**	.21*	.24**	.27**	.22**	.26**	.20*	.144	.20*	.81**	
19. Openness to experience	-.12	-.26**	-.04	.06	.30**	.46**	-.04	.23**	.16	.24**	-.03	-.04	-.01	.44**	.49**	.26**	.78**	.28**

Note. *n* = 140

*p < .05

**p < .01.

^a^Gender was a dichotomous variable (0 = female; 1 = male).

Gender correlated positively (albeit weakly), in favor of males, with the self-assessed visuo-spatial inclinations, but not with objective visuo-spatial abilities (see Table 3).

Although no correlations emerged between objective visuo-spatial abilities and major personality traits, small negative correlations came to light between the objective visuo-spatial abilities and the facets Dynamism, Politeness, and Openness to Culture. Self-assessed visuo-spatial inclinations correlated positively with almost all personality traits and facets (see Table 3). In particular, self-assessed visuo-spatial inclinations showed small correlations with: (i) the Agreeableness trait and its Cooperativeness facet; (ii) the Dominance facet of Energy, the Impulse Control facet of Emotional Stability, and (iii) the Openness to Culture facet of Openness. On the other hand, they showed medium correlations with the Conscientiousness trait and its Perseverance and Scrupulousness facets, and with the Emotional Stability trait and its Emotion Control facet.

#### Hierarchical regression analyses with major personality traits as predictors of objective visuo-spatial abilities and self-assessed visuo-spatial inclinations

In the case of the objective visuo-spatial abilities, all the predictors explained 41% of the variance, and the final model was significant, *F*_(7,132)_ = 13.30, *p* < .001. In Step 1, age and gender accounted for a significant, moderate portion of variance (35%, *p* < .001), with a significant contribution only of age. In Step 2, personality traits accounted for an additional significant, but small portion of variance (6%, *p* < .05), with a significant contribution of Conscientiousness (see Table 4).

**Table 4:** Hierarchical regression analyses with age, gender and personality traits as predictors for the composite scores for objective visuo-spatial abilities and self-assessed visuo-spatial inclinations, respectively. *R*^2^, Δ*R*^2^, and standardized β concern each step, while B and 95% CI concern the last step (Model 2).

	Objective visuo-spatial abilities				Self-assessed visuo-spatial inclinations			
	Model 1	Model 2			Model 1	Model 2		
	β	β	B	95% CI	β	β	B	95% CI
Constant			1.11*	[0.09–2.12]			-4.11***	[-5.42 –-2.77]
Age	-.58***	-.63***	-0.02***	[-0.03 –-0.02]	.05	-.00	-0.00	[-0.01–0.01]
Gender^a^	.12	.05	0.07	[-0.13–0.28]	.24**	.21**	0.38**	[0.11–0.65]
ENERGY		-.12	-0.20	[-0.43–0.03]		.06	0.12	[-0.17–0.42]
CONSCIENTIOUSNESS		.15*	0.19*	[0.05–0.38]		.33***	0.53***	[0.28–0.77]
EMOTIONAL STABILITY		.13	0.14	[-0.01–0.29]		.28***	0.36***	[0.16–0.55]
AGREEABLENESS		-.08	-0.12	[-0.35–0.11]		.13	0.20	[-0.10–0.50]
OPENNESS		-.06	-0.08	[-0.29–0.12]		-.01	-0.02	[-0.29–0.25]
*R*^2^	.35***	.41*** .06*			.06*	.31*** .25***		
Δ*R*^2^								

Note. *n* = 140

*p < .05.

**p < .01.

***p < .001.

^a^Gender was a dichotomous variable (0 = female; 1 = male).

As for the self-assessed visuo-spatial inclinations, all the predictors explained 31% of the variance, and the final model was significant, *F*_(7,132)_ = 8.43, *p* < .001. In Step 1, age and gender accounted for a small portion of variance (6%, *p* < .05), with a significant contribution only of gender (see Table 4). In Step 2, personality traits accounted for an additional significant moderate portion of variance (25%, *p* < .001), with a significant contribution coming from Conscientiousness and Emotional Stability (see Table 4).

#### Hierarchical regression analyses with narrower facets of personality as predictors of objective visuo-spatial abilities and self-assessed visuo-spatial inclinations

As regards the objective visuo-spatial abilities, all the predictors explained 46% of the variance, and the final model was significant, *F*_(12,127)_ = 9.12, *p* < .001. In Step 1, age and gender accounted for a significant moderate portion of variance (36%, *p* < .001), with a significant contribution only from age (see Table 5). In Step 2, personality facets accounted for an additional significant, but only modest portion of variance (10%, *p* < .01), with a significant contribution coming from Dynamism, Emotion Control and Politeness (see Table 5).

**Table 5:** Hierarchical regression analyses with age, gender and personality facets as predictors for the composite scores for objective visuo-spatial abilities and self-assessed visuo-spatial inclinations, respectively. *R*^2^, Δ*R*^2^, and standardized β concern each step, while B and 95% CI concern the last step (Model 2).

	Objective visuo-spatial abilities				Self-assessed visuo-spatial inclinations			
	Model 1	Model 2			Model 1	Model 2		
	β	β	B	95% CI	β	β	B	95% CI
Constant			1.20*	[0.17–2.23]			-3.39***	[-5.33 –-2.65]
Age	-.58***	-.61***	-0.02***	[-0.03 –-0.02]	.05	.00	0.00	[-0.01–0.01]
Gender^a^	.12	.02	0.02	[-0.18–0.23]	.24**	.18*	0.31*	[0.05–0.60]
Dynamism		-.21*	-0.23*	[-0.41 –-0.05]		-.07	-0.08	[-0.32–0.15]
Dominance		-.05	-0.06	[-0.23–0.11]		.06	0.09	[-0.13–0.31]
Scrupulousness		.09	0.10	[-0.10–0.28]		.16	0.22	[-0.01–0.45]
Perseverance		.02	0.02	[-0.16–0.20]		.19*	0.25*	[0.02–0.50]
Emotion Control		.22**	0.21**	[0.05–0.36]		.37***	0.41***	[0.21–0.62]
Impulse Control		-.08	-0.07	[-0.23–0.07]		-.06	-0.07	[-0.26–0.13]
Cooperativeness		.18	0.23	[-0.01–0.46]		.22*	0.32*	[0.02–0.63]
Politeness		-.17*	-0.22*	[-0.41 –-0.02]		.01	0.01	[-0.24–0.26]
Openness to Culture		.01	0.01	[-0.15–0.16]		.02	0.03	[-0.17–0.24]
Openness to Experience		-.08	-0.08	[-0.26–0.09]		-.02	-0.04	[-0.27–0.19]
Total *R*^2^	.36***	.46*** .10**			.06*	.37*** .31***		
Δ*R*^2^								

Note. *n* = 140

*p < .05.

**p < .01.

***p < .001.

^a^Gender was a dichotomous variable (0 = female; 1 = male).

When self-assessed visuo-spatial inclinations were considered, all the predictors explained 37% of the variance, and the final model was significant, *F*_(12,127)_ = 6.00, *p* < .001. In Step 1, age and gender accounted for a small portion of variance (6%, *p* < .05), with a significant contribution only of gender (see Table 5). In Step 2, personality facets accounted for an additional significant moderate portion of variance (31%, *p* < .001), with a significant contribution coming from Perseverance, Emotion Control and Cooperativeness (see Table 5).

## Discussion

This study newly investigated in young and older adults whether, and to what extent, major traits and narrower facets of personality–envisaged in the Big Five model [2]—influence visuo-spatial competence in terms of both objective visuo-spatial performance (in tasks involving rotation and VSWM abilities), and self-assessed visuo-spatial inclinations (involving pleasure and anxiety in exploring places).

First, the results of factor analysis confirmed that the visuo-spatial measures considered can be divided into two factors, represented by objective visuo-spatial abilities on the one hand, and by self-assessed visuo-spatial inclinations on the other, consistently with previous evidence [24]. They also confirmed that objective visuo-spatial abilities include rotation abilities and VSWM [24], which respectively represent higher-order visuo-spatial skills and visuo-spatial processing abilities.

Regression analyses enabled us to better clarify the association between personality (traits and facets) and visuo-spatial competence. First, the results confirmed the role of age (in line with the findings emerging from correlations analyses) in predicting a larger part of the variance -than personality traits- in objective visuo-spatial task performance (35%), but not in self-assessed visuo-spatial inclinations. This pattern of results confirms that older people encounter greater difficulties than younger adults in tasks that involve processing and manipulating visuo-spatial information [34,35], while their self-assessed visuo-spatial inclinations do not change over time [26,34].

Gender was found to account for a significant, but small portion of variance in self-assessed visuo-spatial inclinations (in favor of men), in line with previous reports across the adult life span [26], but did not influence objective visuo-spatial task performance (as also shown by the correlation analyses). It is worth noting that the few aging studies on gender-related differences in visuo-spatial task performance either found no gender effects [47] or, at most, found them only modest (by comparison with the effects of age) and dependent on the visuo-spatial task considered [48–50].

More interestingly, beyond the role of age and gender, personality traits (and their facets) predicted both objective visuo-spatial performance and self-assessed visuo-spatial inclinations, albeit to a variable extent.

Concerning objective visuo-spatial abilities, they were only predicted by Conscientiousness, and only weakly. Our pattern of findings is in line with the general notion that higher Conscientiousness is associated with a better cognitive performance—and objective visuo-spatial tasks performance in the case in point [7,8]. This seems to contrast, however, with previous reports of a higher Openness and lower Agreeableness being associated with a better performance in tasks demanding mental rotation [17], and of these two traits, combined with lower Extraversion and lower Neuroticism (i.e., higher Emotional Stability) being associated with a better performance in spatial visualization and reasoning tasks [14–16, 18–20]. Possible explanations for such contrasting findings might relate to the features of the tasks considered here. Unlike those used in previous studies, our tasks had either time constraints (5 minutes for the sMRT and sOPT), or processing demands that increased when participants performed well (all those measuring VSWM). Participants thus needed to be able to organize their time and optimize their resources to complete the task successfully in the former case, or to exert greater attentional control and use more processing resources in the latter. It may be that the greater commitment, organization and persistence characteristic of Conscientiousness helped our participants to perform better in our demanding tasks, overshadowing the role of other major traits, which might be useful in other types of visuo-spatial task with different requirements [14–20].

It is noteworthy that the correlation analyses did not show any significant relationships between objective visuo-spatial abilities and such a major personality trait. This might be because regression analyses, unlike correlation analyses, bring out the significant contribution of traits (and Conscientiousness in particular), after controlling for age and gender, and also for the effects of the other personality traits considered.

Looking specifically at the contribution of the narrower facets of personality, we found -in line with the correlation analyses- that a better performance in objective visuo-spatial tasks was predicted by higher Emotion Control (a facet of Emotional Stability) and Politeness (a facet of Agreeableness)—consistently with previous findings when only the main traits were examined [13–20], and also by lower Dynamism (a facet of Extraversion/Energy). It is worth mentioning that two previous studies involving young and older adults, and seeking a relationship between personality facets and visuo-spatial performance also found lower scores on Assertiveness, which is a facet of Agreeableness [15], and on Depression and Anxiety, which are facets of Neuroticism/Emotional Stability [14,15] associated with a better spatial visualization and reasoning performance. It might be thus argued that performance in tasks demanding the processing and manipulation of visuo-spatial information could be broadly facilitated by being: better able to handle and control negative emotions (have greater Emotion Control); less active and enthusiastic in interpersonal relations (lower Dynamism); less kind and empathic towards other people (lower Politeness); and possibly more focused on investing resources in demanding tasks and in one’s own intellectual achievement than in social interactions [15,20].

The different associations that emerged between personality traits or their narrower facets and objective visuo-spatial performance (i.e., Conscientiousness, but not its facets, or the facets of Emotion Control, Politeness and Dynamism, but not the major traits they reflect) might be due to facets having a specific variance unrelated to the traits they express [10,36,38], and thus making unique contributions to the depiction of personality [10,36–38]. Different associations between personality and cognition (visuo-spatial abilities in our case) might thus emerge when broad domains as opposed to narrower facets of personality are considered. Further, a possible explanation for the different association between personality and objective visuo-spatial performance (as considered here) or fluid intelligence (as considered in previous studies) might also be that the latter are conceived as two distinct sub-factors of intelligence [51,52]. It has already been suggested that different associations between personality and intelligence might emerge when different combinations of hierarchical levels of personality and intelligence are considered [53,54].

It is worth stressing that the contribution of personality in influencing objective visuo-spatial abilities was modest [6,7,17]. It might be that situational factors relating to the tests (e.g., whether feedback on performance is provided), or other individual characteristics (e.g., motivation and engagement [55,56]) have played a part [5,6,57]. It could also be that personality has a role when tasks involve managing meaningful information, unlike laboratory-based visuo-spatial tasks (like those used here), or in more ecological situations [6]. These are only speculations, which would deserve further investigation.

Turning now to self-assessed visuo-spatial inclinations, our regression analyses -in line with the pattern of correlations- showed that higher Conscientiousness and Emotional Stability positively predicted a moderate portion of the variance (25%) in these self-assessed visuo-spatial inclinations, coinciding with higher pleasure in exploring places and lower spatial anxiety. The contribution of narrower personality facets was at least partly similar to those of major traits: higher Scrupulousness (a facet of Conscientiousness), Emotion Control (a facet of Emotional Stability), and also Cooperativeness (a facet of Agreeableness) positively predicted a moderate portion of the variance in self-assessed visuo-spatial inclinations. These findings newly extend also to older adults the previously-reported finding that young adults’ personality traits and facets influence their self-reported visuo-spatial inclinations when exploring and visiting places [30]. Further, unlike our findings for objective visuo-spatial abilities, the facets seemed to better capture the associations between those aspects of the broad personality traits they represent and self-assessed visuo-spatial inclinations [10,36–38]. It could be that individuals’ personality—and commitment, emotional control, kindness and sensitivity towards others in particular—might influence their positive or negative attitudes to exploring, and how they typically engage and interact with the environment from both an affective and a behavioral standpoint [29,58]. Self-reported inclinations could thus be conceived as the expression of personality in environment-related situations.

Despite these interesting findings, some limitations need to be mentioned. Future studies should expand and confirm our results, further exploring the association between personality and spatial cognition, also across the adult life span, using: i) a broad battery of visuo-spatial tasks that tap not only rotation and VSWM abilities (as done here), but also other visuo-spatial skills crucial to daily living, such as spatial visualization and spatial perception [25], ii) other spatial self-reports, such as self-assessed sense of direction, or preferred spatial strategies or navigation aids [29,30]; and iii) tasks that demand large-scale abilities (as route learning, spatial orientation) [30,59], known to decline with age [60], that were not considered here, but are related to visuo-spatial competence [21–24].

Finally, given the influence of personality traits and facets on visuo-spatial competence suggested by our results, a joint assessment of personality and cognitive functioning could have important implications for the detection of potential adverse effects on visuo-spatial competence in older adults, especially those who are frail [61,62]. Slight changes in personality traits (including an increase in neuroticism) have indeed been shown to occur in presymptomatic Alzheimer’s disease (AD), and to characterize the transition from mild cognitive impairment to frank dementia, fostering its negative behavioral and cognitive outcomes [61], with visuo-spatial dysfunctions known to be among the first clinical signs of AD [63].

## Conclusions

In conclusion, our findings suggest that, beyond the role of age and gender, different personality traits and facets have a weak influence on objective visuo-spatial abilities, but a stronger effect on self-assessed visuo-spatial inclinations. Our results also underscore the importance of considering not only personality traits but also their narrower facets to better clarify the association between personality and cognition (or spatial cognition in our study), needed especially in aging studies [7]. How personality impact individuals’ visuo-spatial cognition needs to be further explored, but the present study points to the importance of considering it among the individual characteristics that influence young and older adults’ visuo-spatial competence.

## Supporting information

S1 Table Correlation matrix for measures of objective visuo-spatial abilities and self-assessed visuo-spatial inclinations. (DOCX)
