# Supplementary material for: The influence of personality traits and facets on visuo-spatial task performance and self-assessed visuo-spatial inclinations in young and older adults
Source: PLoS One. 2019 Aug 5;14(8):e0220525. doi: 10.1371/journal.pone.0220525 (PMC6681964; doi:10.1371/journal.pone.0220525)
Supplement: S1 Table — (DOCX) [file pone.0220525.s001.docx]

**S1 Table.** Correlation matrix for measures of objective visuo-spatial abilities and self-assessed visuo-spatial inclinations.

|  | 1 | 2 | 3 | 4 | 5 | 6 |
| --- | --- | --- | --- | --- | --- | --- |
| 1. short Mental Rotations Test | - |  |  |  |  |  |
| 2. short Object Perspective Test | -0.37** |  |  |  |  |  |
| 3. Backward Corsi Blocks Test | 0.40** | -0.48** |  |  |  |  |
| 4. Jigsaw Puzzle test | 0.52** | -0.38** | 0.40** |  |  |  |
| 5. Pathways Span Task | 0.33** | -0.47** | 0.42** | 0.46** |  |  |
| 6. Attitude to Orientation Task scale | 0.20* | 0.04 | 0.01 | 0.10 | -0.09 |  |
| 7. Spatial Anxiety scale | -0.27** | 0.14 | -0.06 | -0.14 | -0.01 | -0.55** |

Note. *n*=140; **p* < .05. ***p* < .01
